# Ultrasound-guided microwave ablation in the treatment of recurrent primary hyperparathyroidism in a patient with MEN1: a case report

**DOI:** 10.3389/fendo.2023.1175377

**Published:** 2023-09-19

**Authors:** Zhoujun Liu, Yueting Zhao, Xue Han, Xin Hu, Yuzhi Zhang, Lan Xu, Guofang Chen, Chao Liu, Shuhang Xu

**Affiliations:** ^1^ Endocrine and Diabetes Center, The Affiliated Hospital of Integrated Traditional Chinese and Western Medicine, Nanjing University of Chinese Medicine, Jiangsu Province Academy of Traditional Chinese Medicine, Nanjing, China; ^2^ Department of Ultrasound, The Affiliated Hospital of Integrated Traditional Chinese and Western Medicine, Nanjing University of Chinese Medicine, Jiangsu Province Academy of Traditional Chinese Medicine, Nanjing, China; ^3^ Department of Endocrinology, The Affiliated Wuxi People's Hospital of Nanjing Medical University, Wuxi, China; ^4^ Key Laboratory of Tradtional Chinese Medicine Syndrome & Treatment of Yingbing (Thyroid Disease) of State Administration of Traditional Chinese Medicine, Jiangsu Province Academy of Traditional Chinese Medicine, Nanjing, China

**Keywords:** multiple endocrine neoplasia, primary hyperparathyroidism, microwave ablation, recurrence, case report

## Abstract

**Background:**

Multiple endocrine neoplasia type 1 (MEN1) is an inherited endocrine syndrome caused by the mutation in the tumor suppressor gene *MEN1*. The recurrence rate of primary hyperparathyroidism (PHPT) in patients with MEN1 after parathyroidectomy remains high, and the management of recurrent hyperparathyroidism is still challenging.

**Case presentation:**

We reported a 44-year-old woman with MEN1 combined with PHPT who was diagnosed through genetic screening of the patient and her family members. After parathyroidectomy to remove one parathyroid gland, the patient suffered from persistent high levels of serum calcium and parathyroid hormone, which returned to normal at up to 8 months after ultrasound-guided microwave ablation (MWA) for bilateral parathyroid glands, suggesting an acceptable short-term prognosis.

**Conclusion:**

Ultrasound-guided MWA for parathyroid nodules may be an effective therapeutic strategy for recurrent PHPT in MEN1 patients.

## Introduction

Multiple endocrine neoplasia (MEN) is a group of disorders characterized by adenomatous lesions in multiple endocrine glands. MEN patients develop tumors or hyperplasia successively in three endocrine glands and/or neuroendocrine tissues. MEN1 usually involves the pituitary, parathyroid, pancreas and other glands. Most of MEN1 patients suffer from primary hyperparathyroidism (PHPT) caused by parathyroid hyperplasia and/or adenoma (1). The incidence of involvement in two or more parathyroid glands by MEN1-related PHPT is remarkably higher than that of non-MEN-1-related PHPT (56% vs. 7%) (2). After surgery for parathyroid glands, MEN1-related PHPT persists or recurs in 14-69% of patients (3, 4), hypercalcemia in 50% at 8-12 years (5), and permanent hypoparathyroidism in 0-50% (3, 4).

Reoperation remarkably increases the incidence of complications in MEN1 patients with recurrent PHPT. Percutaneous ethanol ablation (EA) is a minimally invasive procedure characterized by lower risks of serious events than the conventional open surgery. EA has been implemented in MEN1 patients with recurrent PHPT (6, 7). PHPT and secondary hyperparathyroidism are treated with minimally invasive thermal ablation, including microwave ablation (MWA), radiofrequency ablation (RFA), laser ablation (LA) and high-intensity focused ultrasound (HIFU). However, its application to MEN1 patients combined with PHPT has not been reported. Recently, Han, et al. reported a 52-year-old MEN1 patient complicated with relapsed PHPT received ultrasound-guided RFA with a remarkable recovery (8). In the present study, we for the first time reported a case of MEN1 combined with relapsed PHPT who was successfully treated with ultrasound-guided MWA.

## Case presentation

**Figure 1:**
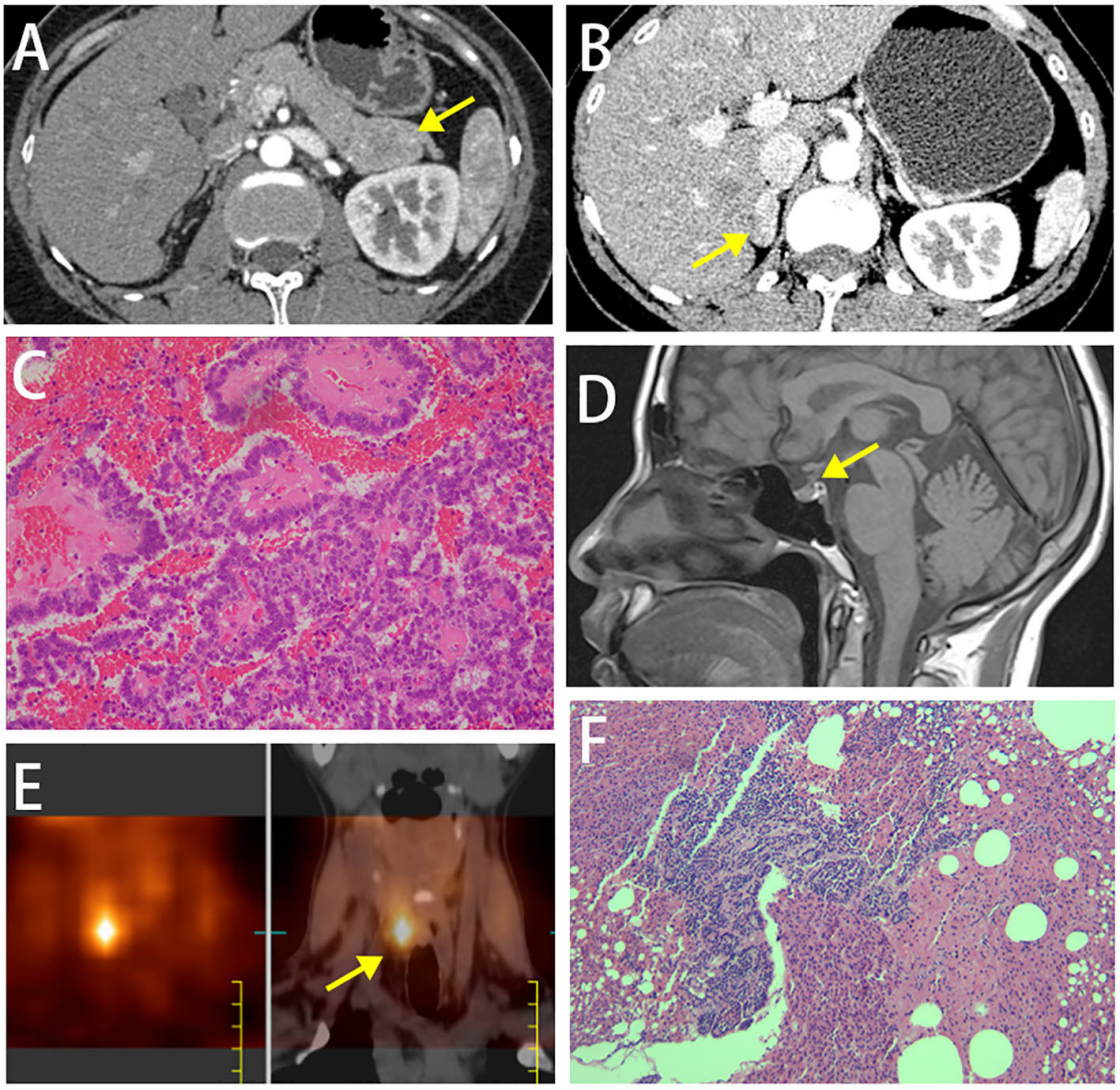
**(A)** Abdominal plain and contrast MRI scans of the pancreas showed a lesion occupying the tail of the pancreas. **(B)** Abdominal plain and contrast MRI scans of the pancreas showed a lesion occupying the right adrenal gland and suspected as adenoma. **(C)** Histopathological image of tissue pancreas tumor removed. **(D)** MRI scan of the pituitary gland. **(E)** Emission computed tomography (ECT) images on parathyroid glands showed one radioactive uptake. **(F)** Postoperative pathology showed nodular hyperplasia of the parathyroid gland.

**Figure 2:**
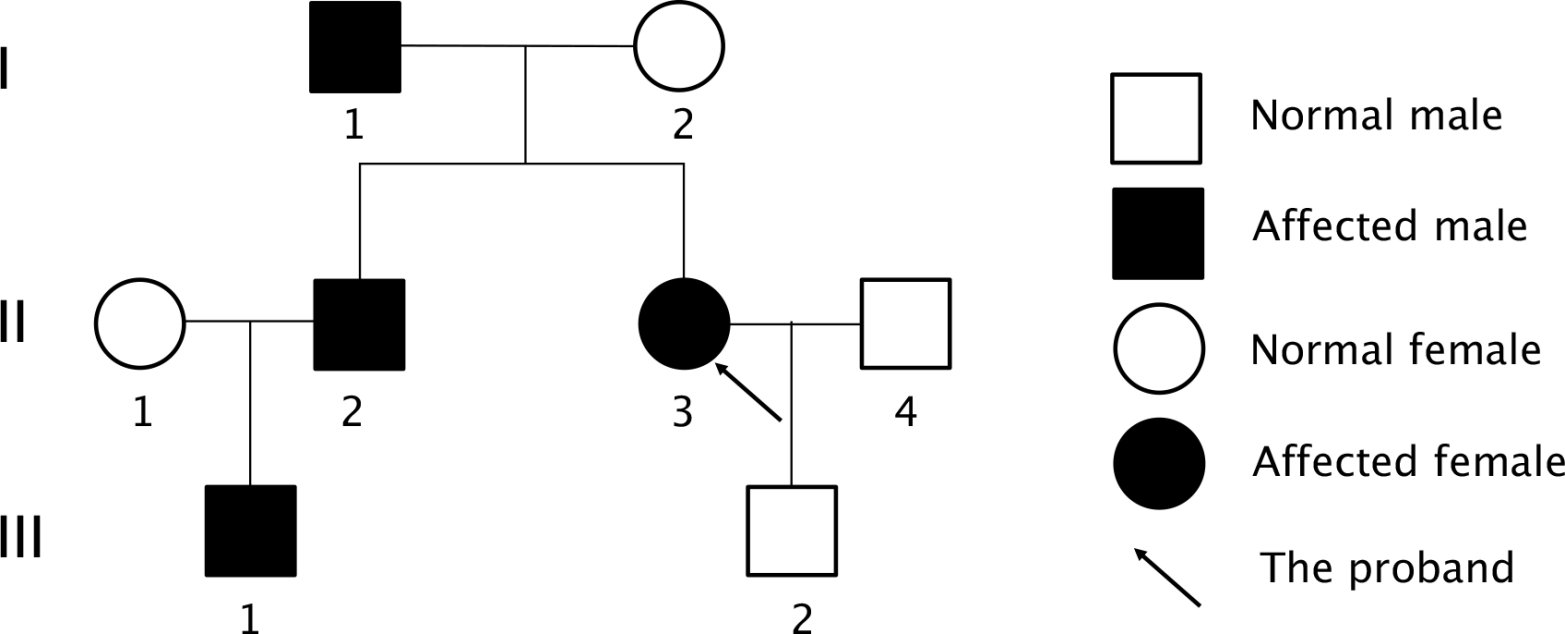
Pedigree diagram of the proband’s family. Family members are indicated by generations (Roman numbers) and individuals (Arabic numbers). Circles indicated women and square indicated men. Clinical status was denoted: open symbols, normal; solid symbols affected.

**Figure 3:**
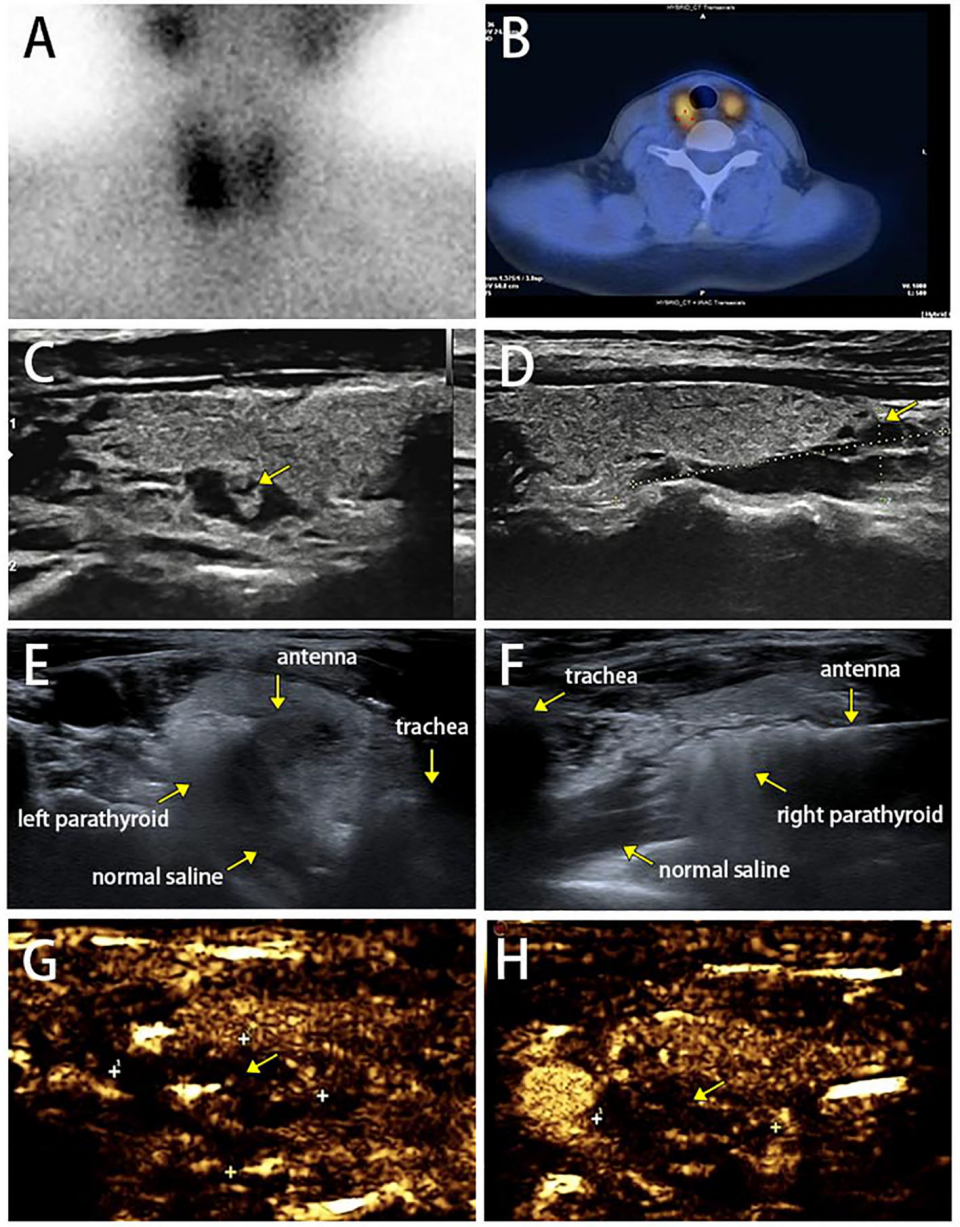
**(A, B)** Dual-phase 99mTc-MIBI parathyroid scan on parathyroid glands with three focal uptakes. **(C, D)** Ultrasound scan of parathyroid glands. **(E, F)** Complete hyperechoic area in the parathyroid glands indicated a complete ablation. **(G ,H)** Postoperative contrast enhanced ultrasound for parathyroid glands did not show blood flow in the remaining lesions.

A 44-year-old woman presented for a pancreatic mass found during the physical examination. Abdominal computed tomography (CT) scan showed: (1) a lesion (38 mm×25 mm) occupying the tail of the pancreas (**Figure 1A**); (2) a lesion occupying the right adrenal gland and suspected as adenoma (**Figure 1B**); (3) small stones in the right kidney. Plain and contrast magnetic resonance imaging (MRI) scans of the pancreas showed: (1) a lesion occupying the tail of the pancreas and suspected as neuroendocrine tumor; (2) the right adrenal adenoma (23 mm×12 mm). The patient received resection of pancreatic body and tail, and the postoperative pathology showed a soft, grayish-yellow mass (4 cm×3cm×2cm) with a clear margin in the tail of the pancreas. Tumor cells in the mass were uniform in size, mildly atypical, and arranged in papillary structure or solid sheets. No tumor cells were found in the resection margin of the tail of the pancreas. Immunohistochemistry results showed AE1/AE3 (+), CgA3 (+), Syn3 (+), Ki67 (about 4%), β-catenin in the membrane (+), α-ACT (+), progesterone receptor (PR, +), and Vimentin (-). G2 neuroendocrine tumor was finally diagnosed based on the pathological and immunohistochemical findings (**Figure 1C**). Serum calcium and parathyroid hormone (PTH) at postoperative 1 month were 2.65 mmol/L and 2847.6 pg/ml, respectively. The patient did not exhibit fatigue, nausea, vomiting, anorexia, abdominal pain, diarrhea, asthma, cough, lower limb pain, thirst, polyuria, constipation, hand and foot convulsions and mental changes during the course of disease.

Thyroid function tests at her age of 28 years were normal. Sex hormone binding globulin serum tests for measuring prolactin level at her age of 30 years were normal. The patient denied the history of medication. She had a healthy son. Her old brother had a medical history of hyperparathyroidism and kidney stones. Physical examinations of the patient showed slight thickening of bilateral toe joints.

Laboratory testing was performed. (1) blood routine tests: white blood cell count, 6.89×10^9^/L; red blood cell count, 3.67×10^12^/L; hemoglobin, 112 g/L; platelet count, 1.0×10^9^/L; (2) biochemical tests: alanine aminotransferase, 40.4 U/L; aspartate aminotransferase, 56 U/L; albumin 36.9 g/L; creatine, 46 µmol/L; creatine clearance, 117.4 ml/min/1.73m^2^; serum sodium, potassium, magnesium and phosphorus levels were normal; (3) urine specific proteins testing: urine α1-microglobulin<4 mg/L; urine IgG, 7.98 mg/L; urine transferrin <2 mg/L; Kappa and Lambda light chains of immunoglobulins in serum and urine were normal; Bence-Jones protein (-); 24-h calcium in urine, 5.55 mmol/L; (4) Multiple times of serum calcium and PTH testing showed abnormal increases (**Supplementary Table 1**).

An uneven signal intensity was detected on the plain MRI scan of the pituitary gland, and no abnormal findings were detected on contrast scans (**Figure 1D**). The dual-phase 99mTc-MIBI parathyroid scan revealed a soft-tissue-density nodule behind the inferior pole of the right thyroid lobe, considering a hyperfunctioning parathyroid mass (**Figure 1E**). Prolactin level of 59.2-120.55 ng/ml, as well as basal prolactin of 63.75 ng/ml and peak/baseline prolactin <1.5 detected by the metoclopramide test were suggestive of hyperprolactinemia. The elevated growth hormone (8.12-10.97 ng/ml↑) and 1-h glucose tolerance test of 8.05 ng/ml indicated the secretion of high-level growth hormone. The aldosterone blood test showed that the orthostatic plasma aldosterone/renin ratio (ARR) was 0.18; 24-h urine catecholamine, 24-hour urine cortisol and cortisol circadian rhythm were in the normal ranges, suggesting the nonfunctional adrenal adenoma. The gastrin level was 28.43 pg/ml↓. Painless gastrointestinal endoscopy showed chronic superficial gastritis with erosions and colonic diverticulum. A normal structure of the right accessory on the gynecological ultrasound scan excluded the possibility of ovarian tumors. A moderate-to-strong echo in the right kidney on the urinary system color Doppler ultrasound was suggestive of the hamartoma. Thyroid function tests and ultrasound did not suggest any thyroid disorder. Z-score of the bone mineral density of L1-4 was below -2.0. CT scan of the chest showed micronodules in both lungs. The patient was finally diagnosed as MEN1 by genetic testing (**Supplementary Figure 1**), revealing a novel mutation c.1520delG>T (p.G507Afs*52) in exon 10 of *MEN1* gene. Moreover, genetic testing was performed in the first-degree relatives of the patient, and the *MEN1* gene mutation was detected in the patient’s father, old brother and nephew (**Figure 2**, **Supplementary Figure 1**).

The patient was treated with the surgical resection of the right inferior parathyroid gland in February 2022 in other hospital, and postoperative pathology showed nodular hyperplasia of the parathyroid gland (**Figure 1F**). Serum calcium and PTH were 2.9 mmol/L and 256.5 pg/ml right after surgery, and 2.78 mmol/L and 294.3 pg/ml at 3 months, respectively, suggesting that hyperparathyroidism was not completely relieved. ECT images on the parathyroid glands in June 2022 showed two adjacent radioiodine uptake tissues (hyperparathyroidism tissues) on the dorsal side of the right thyroid lobe, and one suspected radioiodine uptake tissue on the dorsal side of the left upper thyroid lobe (**Figures 3A, B**). Ultrasound scan of parathyroid glands also showed a hypoechoic nodule in the lower posterior part of the right thyroid lobe (3.1 cm×0.9 cm) and a hypoechoic nodule in the left posterior lobe (0.93 cm×0.46 cm), suggesting benign nodules that may originate from the parathyroid gland (**Figures 3C, D**). The 25(OH)D3 level was 10.1 ng/ml↓, and the Z-scores of BMD in L1-4 were below 2.0.

**Figure 4:**
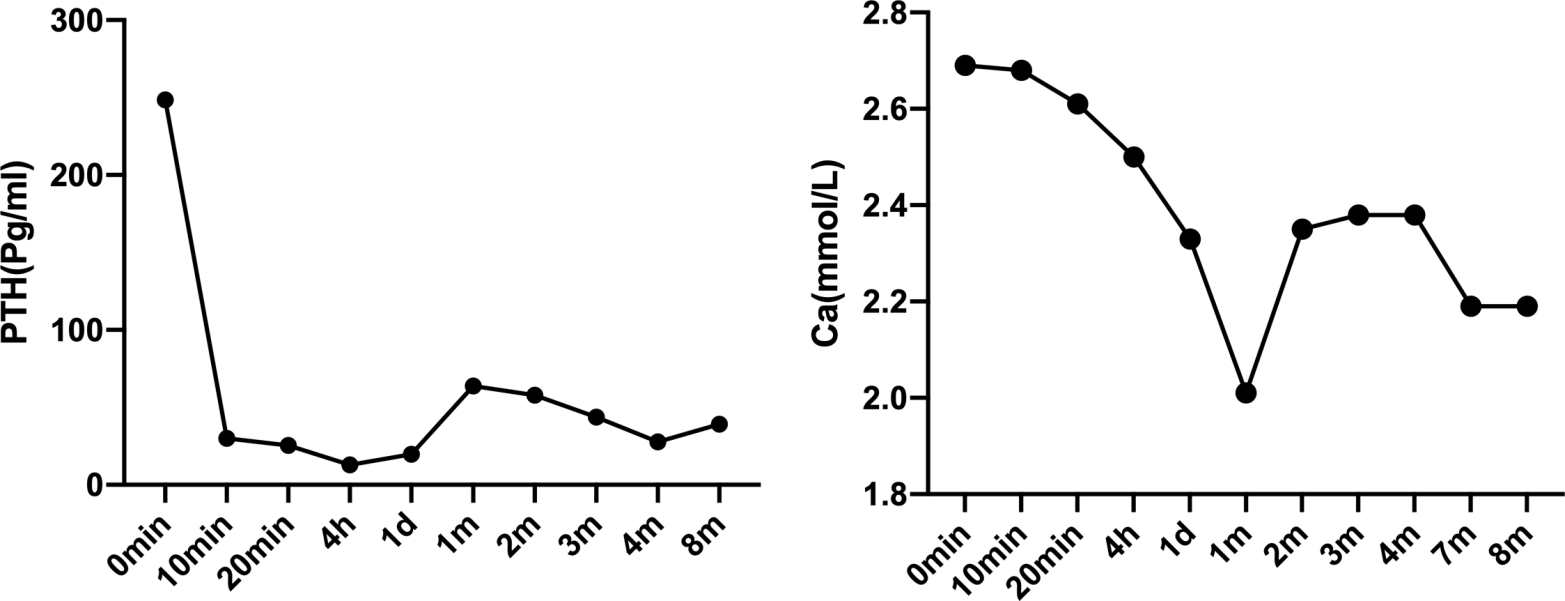
Serum PTH and calcium levels before and after ultrasound-guided MWA. MWA, microwave ablation.

The patient was intervened by MWA for supplementary therapy after being fully informed of therapeutic efficacy and potential risks. The MWA for bilateral parathyroid glands was guided by intraoperative ultrasound, as previously reported (9). The MWA system (KY-2000) was produced by Canyon Medical Inc. (Nanjing, China). Briefly, a total of 78 ml of normal saline was injected and maintained surrounding the parathyroid adenoma to create a barrier that prevents thermal damage to the trachea, esophagus and recurrent laryngeal nerve. The ablation antenna was inserted into the parathyroid lesion through previously determined path, and MWA was initiated at 35 W output power. The operation was sustained until the entire gland was hyperechoic (**Figures 3E, F**). After the ablation, color Doppler ultrasound and contrast-enhanced ultrasound were performed to confirm that no blood flow in the nodule and remaining lesions (**Figures 3G, H**). The MWA procedure lasted 170 s, and intraoperative heart rate, blood pressure and blood oxygen level were 77 beats/min, 136/79 mmHg and 79%, respectively. The patient only complained of mild pain. PTH level immediately returned to the normal at 10 min postoperatively, and remained normal at 20 min, 4 h, 24 h, 1 month, 2 months, 3 months, 4 months, 7 months and 8 months postoperatively, suggesting the complete remission of hyperparathyroidism (**Figure 4**, **Supplementary Table 1**). The patient developed hypocalcemia at 1 month postoperatively due to the irregular supplement of vitamin D and calcium. Serum calcium and PTH levels both returned to the normal after treatment. Postoperative adverse events like obvious hoarseness and hemorrhage were not reported.

## Discussion

MEN1 is a rare syndrome of an autosomal dominant inheritance pattern, which is caused by germline mutations of the *MEN1* gene on chromosome 11q13 (10). The *MEN1* gene, as a tumor-suppressor, encodes menin that regulates gene expressions and cell proliferation by selectively mediating chromatin remodeling (11). Clinical syndromes of MEN1 include PHPT, pancreatic neuroendocrine tumors, and anterior pituitary adenomas. Up to 88-97% of MEN1 patients suffer PHPT due to parathyroid hyperplasia and/or adenoma (1). The incidence of multiple adenomas or hyperplasia in patient with non-MEN1-related PHPT is as low as 7%, while that of involvement of two or more parathyroid glands by MEN1-related PHPT rises significantly to 56% (2).

Currently, surgery is preferred for MEN1-related PHPT. Except for parathyroid malignancies, MEN1-related PHPT is the trickiest in all parathyroid diseases. The rate of persistent or recurrent PHPT ranges 14-69% (3, 4), and the rate of recurrent hypercalcemia is up to 50% at 8-12 years postoperatively (5). Moreover, the incidence of postoperative permanent hypoparathyroidism reaches 50% (3, 4).

Subtotal parathyroidectomy (SPX, removal of 3-3.5 glands)+bilateral cervical thymectomy, total parathyroidectomy (TPX)+bilateral cervical thymectomy+autotransplantation, and less-than-subtotal parathyroidectomy (LPX) are the three most common surgical procedures for MEN1-related PHPT (3). TPX serves as the only curative procedure for MEN1-related PHPT. However, permanent hypoparathyroidism following TPX is a much severer complication than recurrent PHPT. At present, effective methods to assess the viability of autotransplanted parathyroid glands are scant. SPX is an alternative to TPX (12). It is reported that the incidence of permanent hypoparathyroidism following SPX ranges 0-40%, which has not been improved compared with that of TPX (13, 14). A latest meta-analysis revealed that the relative risk of TPX-induced long-term hypoparathyroidism is significantly higher than that of SPX (RR=1.61; 95%CI[1.12-2.31], *P*=0.009) (15). LPX can reduce the incidence of postoperative permanent hypoparathyroidism, and enhance the quality of life (16, 17). Through literature review, the recurrence rate of PHPT in patients with MEN1-related PHPT after TPX ranges 0-60% (3, 18–20). Only one randomized control trial has compared SPX and TPX in 32 MEN1 patients. No significant difference in the recurrence rate is identified (24% vs. 13%, *P*=0.66) (21). Nevertheless, the recurrence rate of PHPT at 19 and 26-28 months of single gland resection (SGE) is 100% (3, 4, 20, 22–24), and the recurrence-free interval after SGE is significantly shorter than that of TPX or SPX (*P*=0.036) (24). A systematic review and meta-analysis compared the recurrence rate of PHPT after SPX and LPX in patients with MEN1-related PHPT, finding that the risks of recurrence, persistence of hyperparathyroidism, and reoperation for hyperparathyroidism after LPX are all significantly higher than those of SPX (15).

Thus, Optimal therapeutic strategies for MEN1-related PHPT that not only possess an acceptable efficacy but also prevent the recurrence and postoperative hypoparathyroidism remain inconsistent. Moreover, the initial time point for parathyroidectomy and the indications of unilateral debridement to young patients with MEN1-related PHPT are controversial (25). In the present case report, the patient was immediately given to the single parathyroidectomy after the diagnosis of MEN1, which remarkably increased the risks of persistent PHPT and reoperation. It is reported that the postoperative persistent PHPT and hypercalcemia in patients with MEN1-related PHPT are significantly linked with anxiety, depression, fatigue, and decreased social function, all deleterious to the quality of life (26).

Current therapeutic strategies for recurrent MEN1-related PHPT include reoperation, EA and medication of calcimimetics (cinacalcet). Reoperation for PHPT largely increases the risks of recurrent laryngeal nerve palsy and permanent hypoparathyroidism (27). For patients with recurrent MEN1-related PHPT who do not meet surgical indications or refuse to be operated, cinacalcet can only maintain normal ranges of serum calcium and PTH, not reduce the risks of complications like fractures and kidney stones (28).

EA is a minimally invasive procedure that is widely applied to patients with non-MEN1-related PHPT (6, 7), showing a high response rate in previous case reports (7, 29). In a prospective study involving 39 patients with inoperable parathyroid adenomas treated with EA, there are 8 (20.5%), 4 (10.3%) and 1 patient (2.6%) receiving 2, 3 and 4 injections, respectively. After 1-month treatment, 46% of them have improved serum PTH and calcium levels, which increases to 84.5% at 1-year follow-up. No severe complications are reported (30). Veldman et al. performed a total of 41 times of EA to 22 patients with residual and recurrent MEN1-related PHPT in 2008 (31). Among them, 82% of patients with initial hypercalcemia successfully return to the normal range of serum calcium or develop hypocalcemia after EA, and no severe complications like permanent recurrent laryngeal nerve injury are reported. Persistent hypocalcemia is only observed in one patient at 12 months postoperatively. The incidence of hypoparathyroidism after EA is 4.5%, which is lower than that in patients with recurrent MEN1-related PHPT after reoperation. Singh et al. assessed the safety and efficacy of EA on patients with recurrent PHPT and MEN1 diagnosed at Mayo Clinic from 1977 to 2013, including 37 patients for 80 times of EA (123 ethanol injections). A normal range of serum calcium is observed after 54 (73%) times of EA, which persists for an average of 24.8 months. Six (8.1%) patients develop postoperative hypocalcemia, and only 4 (5%) suffer the transient hoarseness (32).

Thermal ablation, including MWA, RFA, LA and HIFU, has been applied to treat PHPT, but never MEN1. It is reported that the volume reduction rate (VRR) of PHPT after MWA has considerably reached 79.8-100% (33), and the cure rate, which is defined as the recovery of serum calcium and PTH, ranges 80.0-100.0% (34, 35). The therapeutic efficacy of MWA on PHPT is comparable to that of surgery. The success rate of MWA in the treatment of PHPT is low in some reports, ranging 62.5-63.6% (9, 36). In addition, the incidence of complication of MWA (6.7%) is lower than that of surgery (35). In our previous study, we assessed PTH, serum calcium and VRR in 20 PHPT patients treated with ultrasound-guided MWA from May 2019 to March 2021, and the technical and clinical success rates were 100% and 63.6%, respectively (9). All PHPT-related symptoms were cured after MWA, and severe complications like permanent nerve damage and permanent hypoparathyroidism were not reported. In the present case report, the patient’s serum calcium and PTH recovered immediately after ultrasound-guided MWA. However, postoperative transient hypocalcemia occurred, due to the lack of timely supplementation of vitamin D and calcium, but subsided after a supplementation therapy. The patient has been followed up for closely monitoring serum calcium and PTH. Therapeutic efficacy of EA is linked with the injection method, injection range and ethanol dosage, which usually achieves an acceptable outcome by multiple injections after postoperative evaluations (30, 32). Theoretically, the safety and efficacy of MWA are higher than those of EA, which may be attributed to the complete destruction of the thyroid glands.

MEN1 may bring with other tumors, including adrenal tumor, gastric tumor, skin tumor, subcutaneous tumor, and recently reported breast cancer. Imaging evidence for pituitary tumor in this patient lacked, and corresponding treatment for elevated prolactin and growth hormone levels was not given. In addition, the possibility of breast cancer was not assessed. The patient was postoperatively followed up for less than half a year, and she was still being followed up to analyze long-term efficacy of our treatment.

## Conclusion

Genetic testing of *MEN1* gene and screening of other neuroendocrine tumors were performed for this patient, due to the development of multiple parathyroid adenomas. Surgery is the first-line treatment for MEN1-related PHPT, while postoperative recurrence and reoperation rates are relatively high. Ultrasound-guided MWA is a promising option for MEN patient with recurrent PHPT. A large-scale study with a long-term follow-up is needed in the future, aiming to assess the long-term efficacy and safety of MWA on recurrent PHPT in MEN1 patients.

## Patient perspective

After I was diagnosed with MEN1, I searched related medical knowledge to understand what disease it was and find what treatment would be the best. After undergoing pancreatic surgery, I was further confirmed to have hyperparathyroidism. The first ECT revealed a parathyroid adenoma on the right side of the neck. Then I turned to an experienced surgeon, but only had this parathyroid lesion removed. Unfortunately, soon after surgery, it found that parathyroid hormone and blood calcium did not return to normal. Before Dr. Xu performed microwave ablation for me, I was informed that thermal ablation had been used for the treatment of hyperparathyroidism; however, there is still no report of MEN1 complicated with hyperparathyroidism receiving thermal ablation. Based on his experience in microwave treatment of parathyroid glands and the current literature of EA treatment for MEN1 hyperparathyroidism, he believed that these two parathyroid lesions could be destroyed by microwave ablation to restore parathyroid function.

The microwave ablation process was generally smoother and safer than I thought, except for minor pain and neck swelling. Postoperative monitoring of parathyroid hormone and serum calcium found that both returned to normal quickly. After the operation, I also developed tetany. Treated with vitamin D and calcium supplements, these symptoms were relieved. I hope my experience can help those MEN1 patients. I also hope that doctors can carry out similar research to find a treatment method with less trauma and better efficacy for patients with MEN1 complicated with primary hyperparathyroidism.

## Data availability statement

The data presented in this study will be made available by the authors upon request, without undue reservation.

## Ethics statement

Written informed consent was obtained from the individual(s) for the publication of any potentially identifiable images or data included in this article.

## Author contributions

Patient management: XHa, XHu, YtZ, GC. Performing MWA: SX. Data collection: ZL, XHa, LX. Writing manuscript: ZL. Writing review and editing: CL, YzZ, SX. Supervision: SX, CL. All authors contributed to the article and approved the submitted version.

## Supplementary material

Supplementary Figure 1 Genetic screening of the *MEN1* gene in the proband and her relatives. The arrow indicates the mutation c.1520delG>T (p.G507Afs*52) in exon 10 of the *MEN1* gene. **(A)** the proband. **(B)** the proband’s son. **(C)** the proband’s nephew.
