# Supplementary figures and images for: Ultrasound-guided microwave ablation in the treatment of recurrent primary hyperparathyroidism in a patient with MEN1: a case report
Source: Front Endocrinol (Lausanne). 2023 Sep 19;14:1175377. doi: 10.3389/fendo.2023.1175377 (PMC10546301; doi:10.3389/fendo.2023.1175377)

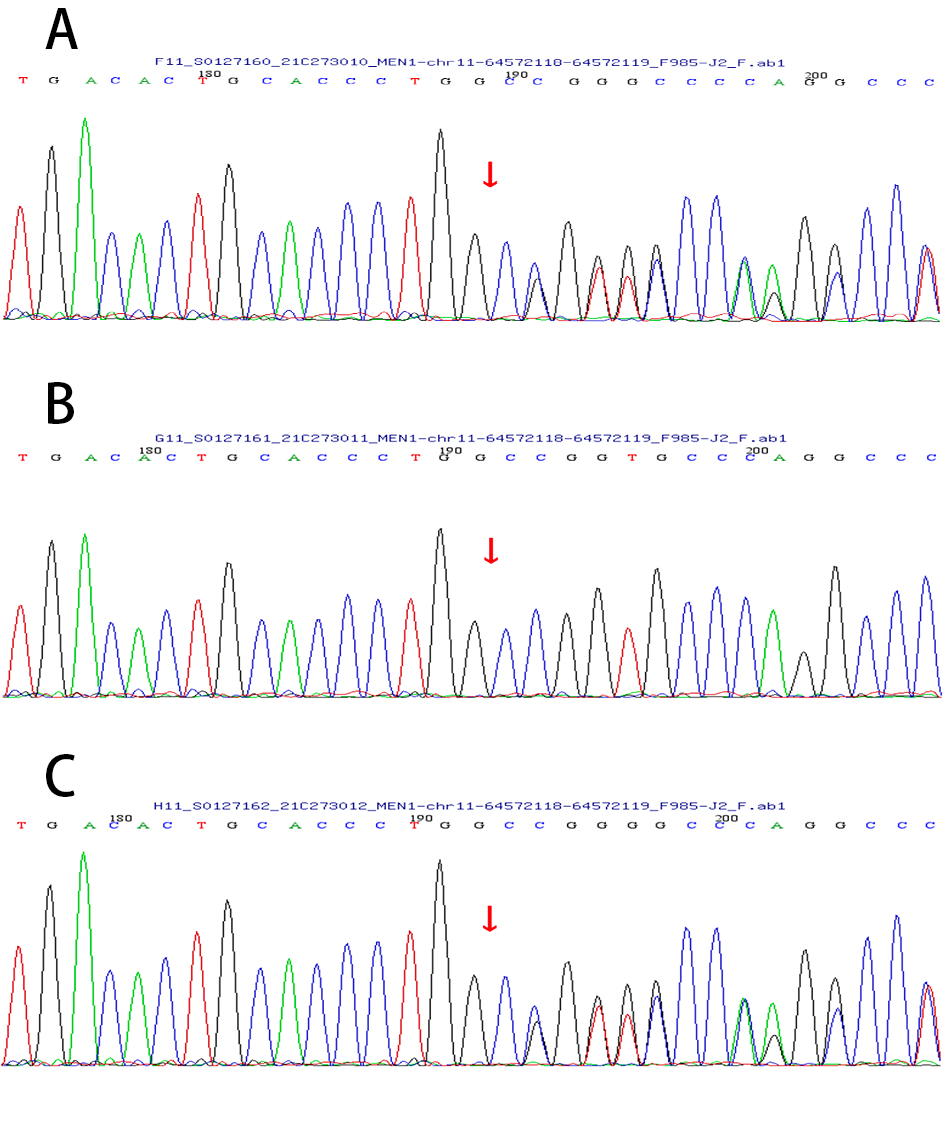

Supplement: Supplementary Figure 1 — Genetic screening of the MEN1 gene in the proband and her relatives. The arrow indicates the mutation c.1520delG>T (p.G507Afs*52) in exon 10 of the MEN1 gene. (A) the proband. (B) the proband’s son. (C) the proband’s nephew. [file Image_1.tif]
