# Supplementary material for: Ultrasound-guided microwave ablation in the treatment of recurrent primary hyperparathyroidism in a patient with MEN1: a case report
Source: Front Endocrinol (Lausanne). 2023 Sep 19;14:1175377. doi: 10.3389/fendo.2023.1175377 (PMC10546301; doi:10.3389/fendo.2023.1175377)
Supplement: Supplementary file 2 [file Table_1.docx]

Supplementary table 1. Monitoring of serum calcium, PTH and 25(OH)D3.

| Date | Serum calcium (mmol/L) | PTH (pg/ml) | 25(OH)D3 (ng/ml) |
| --- | --- | --- | --- |
| 2022.1.7 | 2.65 | 2847.6 |  |
| 2022.1.17 | 2.69 | 2592 |  |
| 2022.1.26 | 2.72 | 1071 |  |
| 2022.2.09, 2^nd^ day after PX | 2.90 | 256.5 |  |
| 2022-05-07 | 2.78 | 294.30 |  |
| 2022-06-14 | 2.7 | 218.8 | 10.1 |
| 2022-06-17, MWA |  |  |  |
| 0min | 2.69 | 248.60 |  |
| 10min | 2.68 | 30.02 |  |
| 20min | 2.61 | 25.47 |  |
| 4h | 2.5 | 12.79 |  |
| 2022-06-18 | 2.33 | 19.65 |  |
| 2022-06-20 | 2.07 | 22.97 |  |
| 2022-07-02 | 2.01 | 63.80 |  |
| 2022-07-19 | 2.03 | 70.20 | 11.22 |
| 2022-08-15 | 2.35 | 57.90 | 16.89 |
| 2022-09-09 | 2.38 | 43.70 |  |
| 2022-10-05 | 2.38 | 27.80 | 18.26 |
| 2023-01-08 | 2.19 |  |  |
| 2023-02-12 | 2.19 | 39.10 |  |

PTH, parathyroid hormone. PX, parathyroidectomy. MWA, microwave ablation.
